# The role of mental accounting in risk-taking and spending: a meta-analysis of the house-money effect

**DOI:** 10.3389/fpsyg.2025.1549626

**Published:** 2025-07-03

**Authors:** Kasumi Dan

**Affiliations:** Graduate School of Economics, Keio University, Tokyo, Japan

**Keywords:** mental accounting, house-money effect, windfall, risk taking, decision making, systematic review, meta-analysis

## Abstract

**Introduction:**

This study systematically analyzes the house-money effect, a phenomenon in which people become more financially risk-taking and wasteful after receiving unexpected income. It aims to identify the general tendencies and factors that influence this effect, because the results reported in previous studies are inconsistent.

**Method:**

A total of 36 eligible studies with 57 continuous and 18 binary outcome effect sizes were included in this meta-analysis. A random-effects model was used to pool the effect sizes.

**Results:**

A low-to-moderate house-money effect (g = 0.37, rr = 1.33) was confirmed. However, high heterogeneity was observed, and the strength of the house-money effect varied widely, depending on the situation. The subgroup and meta-regression analyses revealed several moderators. While a strong effect was observed in the controlled experimental environment, the effect was weakened when it was closer to a real-world environment. For continuous outcomes, the effect was particularly pronounced in students and Asian regions, and the effect size decreased as the publication year increased, suggesting the limited universality of the house-money effect. In the publication-bias analysis, a slight bias was detected using multiple methods.

**Discussion:**

This suggests that the true effect size may be smaller, supporting the theory that the house-money effect is reproducible only under certain conditions.

## 1 Introduction

Money is inherently fungible, and its value should remain the same regardless of the source from which it is obtained. In principle, money should be valued the same regardless of the means by which it is obtained, as long as the amount is the same. However, the theory of mental accounting argues that people often behave in ways that contradict such rational economic principles. Mental accounting explains how people psychologically label, categorize, and “color” money, treating the same amount as having different characteristics (Thaler, 1985). In other words, people change how they spend money depending on the context in which it was received, even if the amount is the same. A common example is the house-money effect, which refers to changes in spending behavior based on the source of income, demonstrating a classification consistent with mental accounting. The house-money effect refers to people being more generous with unexpected or unearned income than ordinary income or savings (Clark, 2002). It also describes increased financial risk-taking after a windfall (Thaler and Johnson, 1990). One illustrative example of the former is the study of Epley et al. (2006), in which students received a tuition refund from their university. Those who were told the refund was a “bonus” spent more of the money than those who were told it was a “rebate.” Although the amount and source were identical, students who perceived the refund as an unexpected gain—essentially a form of additional income—tended to spend it more freely, whereas those who understood it as a reduction of previously paid tuition, and thus simply the return of their own money, were more inclined to save it (Epley et al., 2006). Carlsson et al. (2013) illustrated a similar tendency: people who received money without doing anything donated more than those who received it as a reward for performing a task. In other words, those who obtained money easily were also more willing to part with it easily. This shows that people tend to treat money earned through their own effort more cautiously, whereas money gained effortlessly is more readily spent (Carlsson et al., 2013). In line with the latter definition, people who had just won a gamble and gained some monetary profit were more likely to choose an additional high-risk gamble, and this behavior is similar to how someone who wins the lottery may continue purchasing more tickets, while someone who loses tends to stop (Thaler and Johnson, 1990). The house-money effect effectively explains a decision-making bias where people classify, and label money based on its source and treat it as having different characteristics (Thaler, 1999). This is a prime example of how mental accounting influences risk-taking and spending behavior. Therefore, this study focuses specifically on the house money effect.

The house-money effect is an important everyday phenomenon that has been studied for over 30 years. Indeed, research has found this effect to vary across studies. Some report a significant increase in spending with windfall income compared to ordinary income (Carlsson et al., 2013; Houser and Xiao, 2015; Peng et al., 2013), while others show a weaker house-money effect (Dannenberg et al., 2012; Stivers et al., 2020). Moreover, a “reverse house-money effect,” where people reduce spending or become risk-averse after receiving a windfall, has been observed (Juergensen et al., 2018; Cho et al., 2023). Even in the same experiment, subgroups have shown stronger and reverse effects, depending on individual attributes and cognitive tendencies (Hackinger, 2024), with the strength of the effect likely depending on factors such as environment, methods, and participant characteristics.

However, to the best of our knowledge, no systematic analysis has been conducted, and the results of previous studies were inconsistent. This study provides the first comprehensive systematic review, identifying generalizable trends and influencing factors, making previous findings more applicable. Further, this study positions itself within the previously conflicting context as research that offers a provisional conclusion on the existence of the house-money effect. That is, it aims to determine the general tendency and robustness of the house-money effect, and identify the factors influencing its effect size. Previous research included both continuous and binary outcomes. However, effect size calculations differ for these two outcomes. Since integrating effect sizes with different units and definitions is meaningless, continuous and binary outcomes are analyzed separately. The process involves examining overall trends, performing subgroup analyses, then advancing to meta-regression, and finally, assessing publication bias.

This meta-analysis reveals a consistent pattern across prior studies: financial risk-taking and spending behavior tend to increase when the money involved is unexpected or unearned. However, this effect is far from universal. It appears stronger among students and in Asian countries, but fades in real-world settings outside the lab. Interestingly, more recent studies have reported weaker effects, suggesting that this once widely accepted psychological bias may be limited to specific situations.

## 2 Materials and methods

### 2.1 Search strategy

This review was not registered because it is an exploratory study, and registration was not deemed necessary for its design and purpose. This study followed the Preferred Reporting Items for Systematic Reviews and Meta-Analyses (PRISMA) guidelines (Page et al., 2021) (The PRISMA checklist is included in the Supplementary material). The rationale for adopting an exploratory approach was that a preliminary review of past literature revealed significant variability in findings, making it difficult to specify hypotheses in advance. Accordingly, the lack of pre-registration allowed for analytical flexibility, which was employed to apply appropriate analytical strategies in response to the observed data patterns. Such flexibility was exercised in accordance with established norms for meta-analysis, including the PRISMA guidelines. Therefore, while the absence of a pre-registered protocol may warrant caution, we believe it does not substantially compromise the methodological transparency or replicability of this study.

Searches were conducted in Web of Science and Scopus using the keywords “house money effect” and “windfall,” entered separately without the use of explicit Boolean operators such as AND or OR. Web of Science was searched in all fields, and Scopus in article title, abstract, and keywords. The final search was performed on November 14, 2024. Gray literature was excluded because of the difficulty in accessing all such sources, the risk of arbitrary selection, and to ensure high study quality. The screening process was conducted with expert consultation and oversight to avoid errors and inappropriate biases. The initial search yielded 5,031 results. Of these, 130 articles that could not be imported into the Mendeley reference management software because of inaccessible bibliographic information or content, were excluded. In addition, 1,186 duplicates were removed. Of the remaining 3,715 articles, 3,620 unrelated to the house-money effect were excluded after reviewing the abstracts or content, which included many topics such as house prices or oil windfalls.

### 2.2 Eligibility criteria

After the exclusion, 95 articles were screened. The eligibility criteria were based on the population, intervention, control group, and outcome (PICO) framework: (1) Studies that measured spending or financial risk-taking related to windfalls and unexpected or unearned income; (2) Empirical studies; (3) Studies that used a between-subjects design with a control group; (4) Studies that focused on individual decision-making; and (5) Studies in which the control group received no or low-cost benefits.

**Figure 1:**
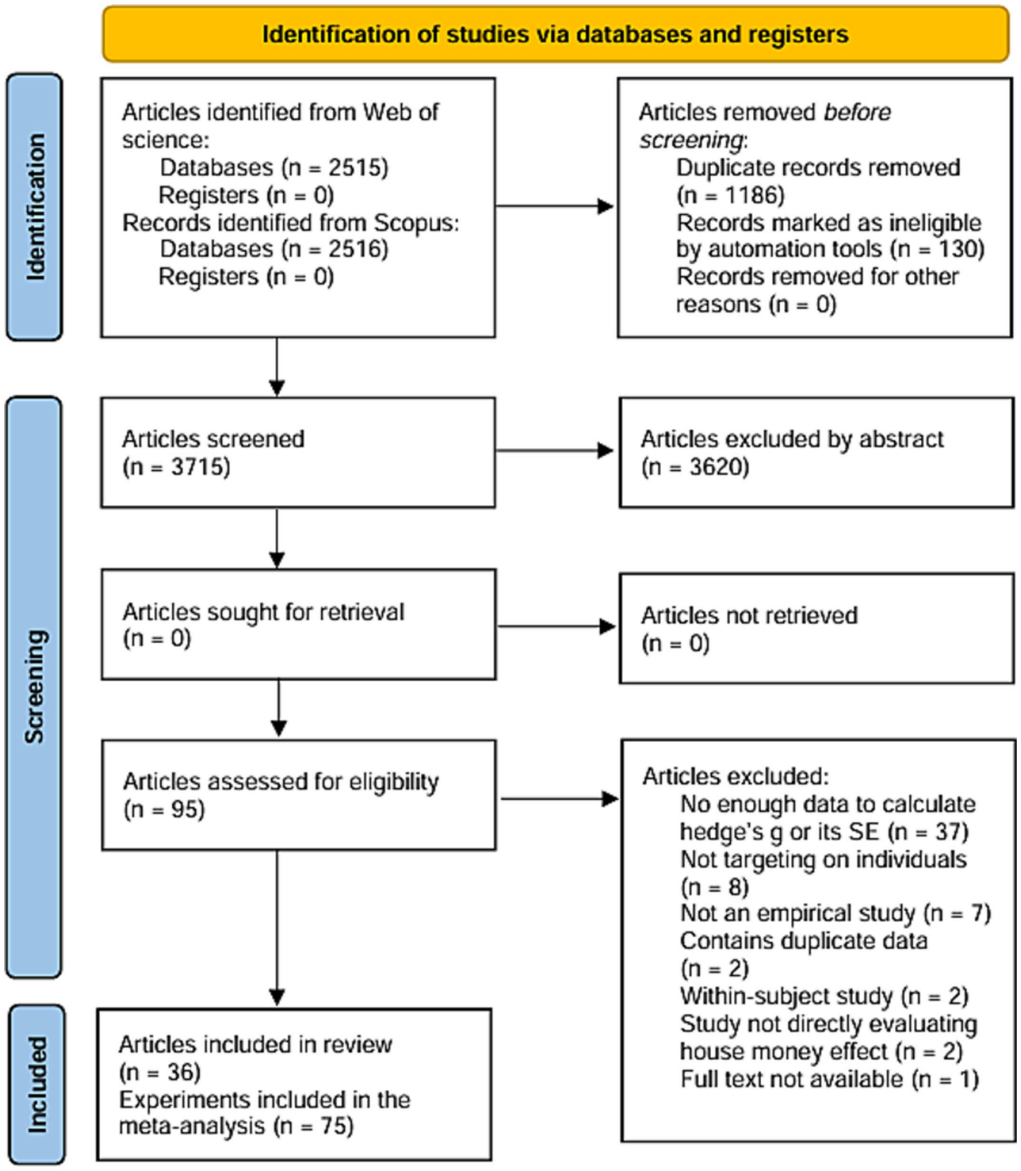
Study selection flow diagram. For example, the study by Fonseca and Rahimi (2022) included in “Study not directly evaluating house money effect” measured tax return compliance rates for both windfall income and regular income. However, tax compliance is a situation that is quite different from regular expenditures, and we therefore considered that even if something like a house money effect was observed, it should not be explained as “waste” after windfall income, and therefore we excluded it. Studies that met multiple reasons for exclusion were counted for the most primary reason.

Articles that did not meet these criteria, including theory building and review articles, books, and studies that focused on firm or household decisions, were excluded. Additionally, 37 articles were excluded because of insufficient information to calculate Hedge’s g and the standard error. Figure 1 shows further reasons for exclusion. Four studies included effect sizes calculated using additional information obtained from the authors. From the remaining 83 effect sizes, studies with non-independent participants were combined, resulting in 75 effect sizes from 36 articles: 57 with continuous outcomes and 18 with binary outcomes.

### 2.3 Data extraction

Extracted data included sample sizes for the control and treatment groups, mean and standard deviation of expenditure (or its indicator), publication year, study design, participant country, age group, and data required to calculate the risk ratios (for binary outcomes). Studies that reported only regression coefficients and standard errors were used as mean differences. When means were unavailable, *t*-values or *χ*^2^ values were extracted if provided. The data extraction was verified primarily by the author, with expert consultation to ensure accuracy and eliminate potential biases. Details of the included studies and extracted data are shown in Supplementary Figures 1, 2, respectively.

### 2.4 Statistical procedure

For studies with continuous outcomes, Hedge’s g (standardized mean difference with a small sample bias correction) was used. For issues regarding units of analysis, such as multiple treatment groups sharing one control group, we combined the groups using the dmetar package in R (Mattos and Ruellas, 2015). For binary outcomes, we followed Harrer et al. (2021), preferring risk ratios over odds ratios. They noted that odds ratios are often confused with risk ratios, which can lead to misinterpretation of the magnitude of effects. When unit of analysis issues occurred, sample sizes and events were averaged into a single effect size.

If effect sizes could not be calculated from the averages, *t*-values or regression coefficients were used using the esc package (Lüdecke et al., 2019). We pooled effect sizes using a random effects model via the meta package (Balduzzi et al., 2019), estimating $\tau^{2}$ with the maximum likelihood method for continuous outcomes and the Paule-Mandel method for binary outcomes, as per Veroniki et al. (2016). Sensitivity analysis was used to check outputs of different methods. The weights, effect sizes, and 95% CIs appeared in the forest and Drapery plots with *z*-tests for significance. Heterogeneity indices included Cochran’s Q, $I^{2}$ , H, and $\tau^{2}$ . Outliers and influence diagnostics were assessed using the metafor (Viechtbauer, 2010) and dmetar packages. Outlier analysis is based on the 95% confidence interval.

A general analysis of all eligible effect sizes was performed first. In addition, a multilevel meta-analysis was conducted. Subsequently, subgroup analyses with the meta-package using a fixed-effects multiple model (mixed-effects). Following Borenstein et al. (2011), common $\tau^{2}$ estimates were used for subgroups with ≤5 effect sizes; otherwise, separate estimates were applied. Subgroup analyses identified variables that influenced the effect size, which were subjected to meta-regression analyses for further verification. Following Higgins and Thompson (2004), we checked the robustness of the models using permutation tests. Publication bias was assessed using a combination of funnel plots, Egger’s test, trim-and-fill, Limit meta-analysis, and *p*-curve analysis to ensure robust results considering high heterogeneity. The analyses were performed using the meta, metasens (Schwarzer et al., 2020), and dmetar packages. Each plot was generated using Tidyverse (Wickham et al., 2019). The significance level was set at 5% for all analyses. Although there is no protocol for this systematic review, anyone can replicate this study using the Supplementary Figure data on the included studies and the R packages.

Statistical power was examined using two complementary approaches: a parametric and a non-parametric analysis. First, we conducted a parametric power analysis under a random-effects model using the dmetar package (Harrer et al., 2021), which assumes asymptotic normality of the standardized mean difference (SMD), and a significance level of *α* = 0.05. We specified “high” heterogeneity, which in this package does not correspond to a fixed τ^2^ value, but adjusts the standard error based on formulas by Hedges et al. (2010). Under these assumptions, even with only 10 comparisons (each yielding one effect size) and an average sample size of 50 participants per group, the power to detect a moderate pooled effect size (SMD = 0.5) reached 99.9%. Furthermore, the power to detect a small pooled effect size (SMD = 0.2) was 88.3% under the following conditions: (1) 10 comparisons with an average of 100 participants per group, or (2) 20 comparisons with an average of 50 participants per group. These findings suggest that sufficient statistical power is secured even under conservative assumptions, indicating that the present meta-analysis is unlikely to suffer from issues related to statistical power. Second, we conducted a non-parametric power analysis based on the method proposed by Banerjee (2020), which does not rely on distributional assumptions. To calculate the required sample size, we applied the following conservative assumptions: a small effect size (SMD = 0.2), a significance level of *α* = 0.05, and 90% power (*β* = 0.10). Given that our analysis is based on standardized mean differences (SMD), we assumed the variance of the underlying measure to be 1, which is standard when the data have been standardized. Under these assumptions, the required sample size was estimated to be approximately 1,457 participants. These findings suggest that sufficient statistical power is secured even under non-parametric, conservative assumptions that correspond to relatively small total sample sizes for a meta-analysis, like the parametric approach.

## 3 Results

### 3.1 Results of continuous outcome studies

#### 3.1.1 General meta-analysis

**Figure 2:**
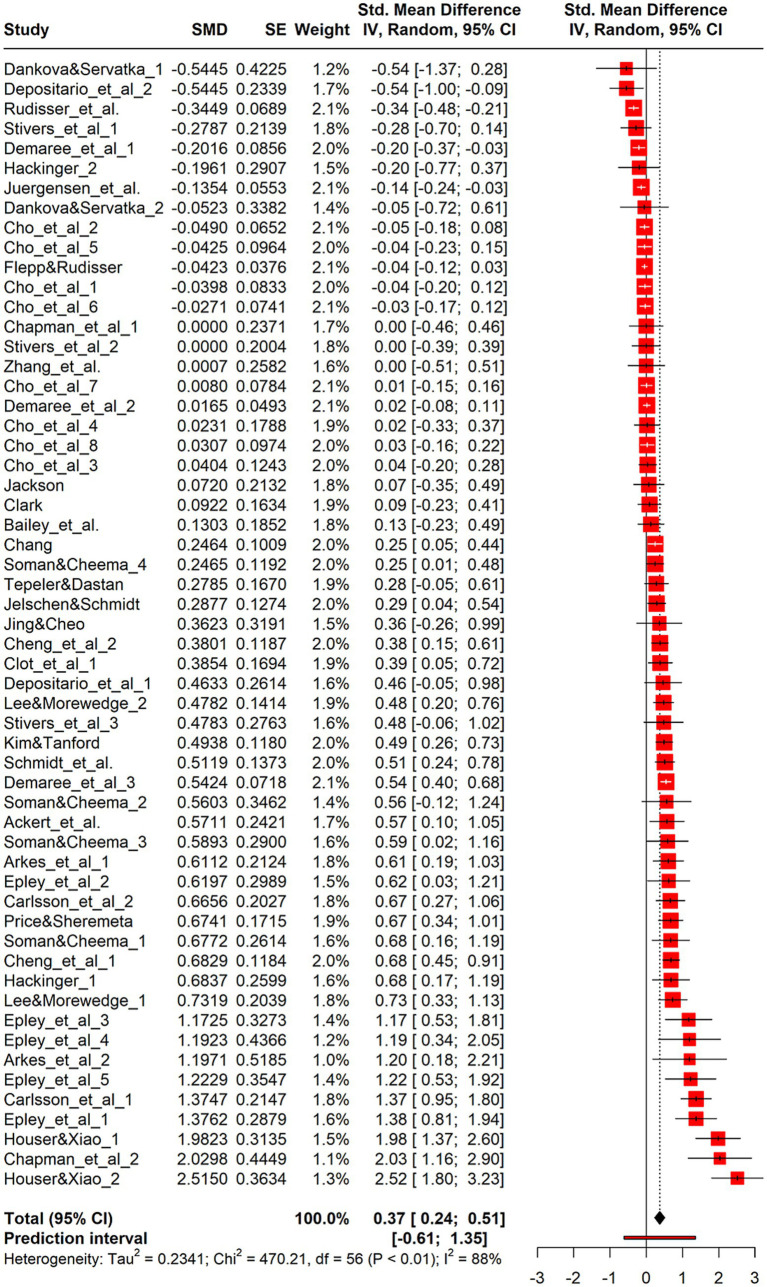
Forest plot for continuous. Forest plot displaying 57 effect sizes from studies with continuous outcome measures (e.g., amount wagered, spending level). The overall pooled effect size is Hedges’ g = 0.38, 95% CI [0.22, 0.55], *p* < 0.001, indicating a moderate house-money effect. Substantial heterogeneity is present, supporting the need for moderator analysis.

The analysis including 57 effect sizes was significant [g = 0.37, 95%CI (0.22, 0.51), *z* = 5.31, *p* < 0.01] (Figure 2 and Supplementary Figure 3), representing a weak to moderate effect as per Cohen (1988). Cochran’s Q (Cochran, 1954) indicated significant heterogeneity [Q (56) = 470.21, *p* < 0.01], justifying the random-effects model. The $I^{2}$ statistic also showed substantial heterogeneity [ $I^{2}$ = 88.1% > 75, 95%CI (85.3, 90.3)] as per Higgins et al. (2002). Furthermore, the confidence interval for $\tau^{2}$ = 0.23 [95%CI (0.24, 0.51)] and H = 2.90 > 1 [95%CI (2.61, 3.21)] did not include zero, indicating heterogeneity (IntHout et al., 2016).

A sensitivity analysis found no significant difference between the Paule-Mandel method (g = 0.38, $\tau^{2}$ = 0.28) and the restricted maximum-likelihood method (g = 0.37, $\tau^{2}$ = 0.24). In this study, meta-analysis was essential because empirical research in social sciences shows varying results, and our goal was to measure the general extent of the house-money effect; therefore, high heterogeneity was not an issue. The prediction interval (IntHout et al., 2016) suggested that effects of future studies could range from −0.61 to 1.35 for g, indicating that windfall income may either reduce risk aversion and spending or significantly increase them.

Several studies contributed multiple effect sizes, introducing statistical dependencies that violate the assumption of independent observations in standard meta-analysis. To address this issue, a three-level meta-analysis was conducted to account for the clustering of outcomes within the same study. Model comparison showed that the three-level model provided a better fit, with lower AIC (85.30 vs. 101.92) and BIC (91.43 vs. 106.01) values than the two-level model. However, the overall effect size estimate remained stable across models: g = 0.36 [95% CI (0.19, 0.53)] under the three-level model, which was only slightly lower and had a modestly wider confidence interval. Variance decomposition indicated that 70.9% of the heterogeneity was due to between-study differences, suggesting that most of the variation occurred at the study level. Given that the core results were robust and to avoid unnecessary model complexity, subsequent analyses were performed using a standard random-effects model.

#### 3.1.2 Outliers and influence analysis

Outlier detection identified 20 outlier effect sizes. Pooling the remaining 37 effect sizes reduced heterogeneity slightly [Q (36) = 74.99, *p* < 0.01, $I^{2}$ = 52.5, 95%CI (30.1, 67.1), $\tau^{2}$ = 0.03, 95%CI (0.01, 0.11), H = 1.44, 95%CI (1.20, 1.74)]. The prediction interval ranged from 0.03 to 0.71 for g. However, the large number of excluded outliers limited the interval’s generalizability.

**Figure 3:**
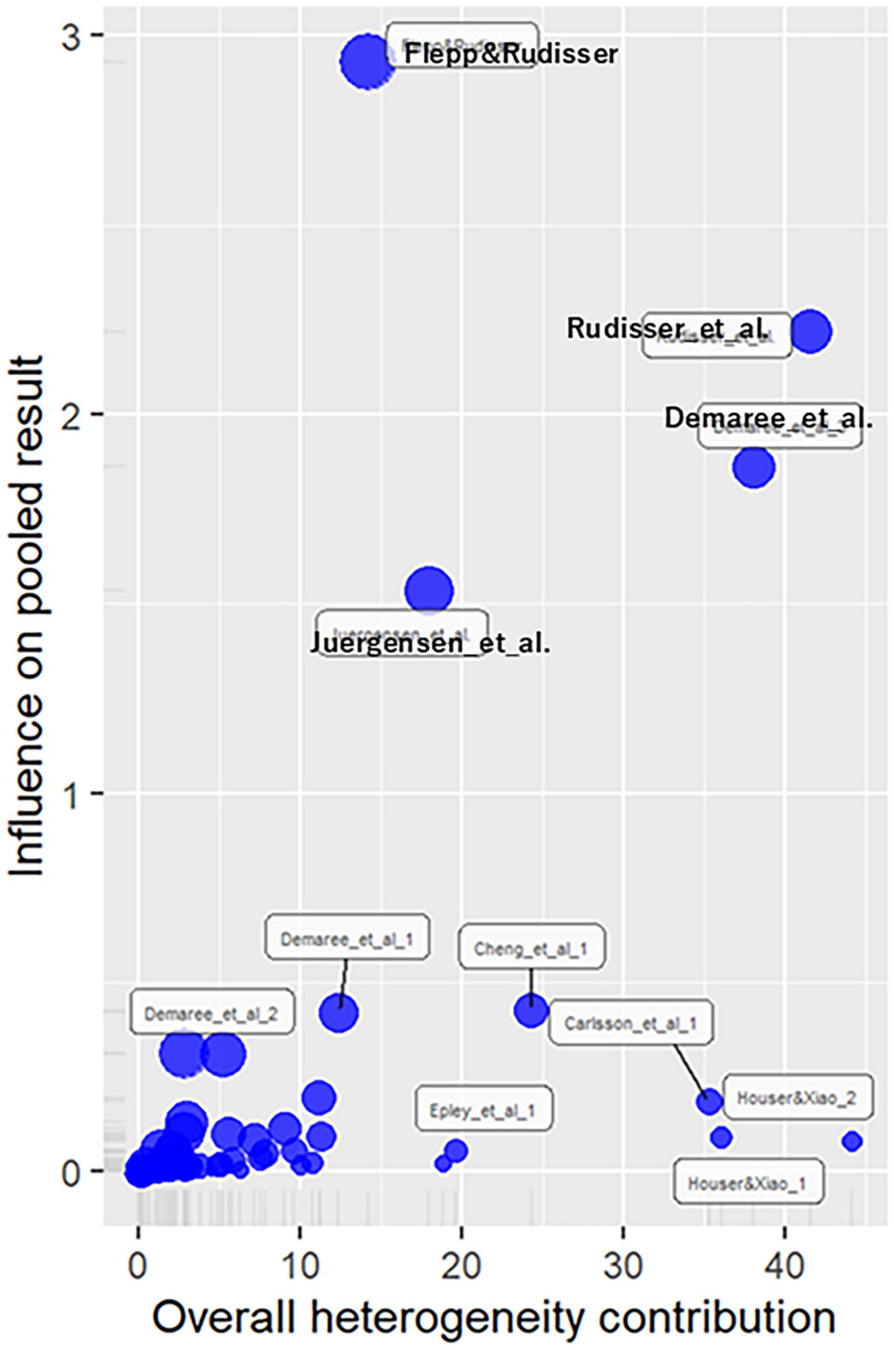
Influence diagnostics plot for continuous outcomes. This plot highlights studies with high influence on the overall effect size. While four effect sizes showed strong influence, their removal had minimal impact on the overall estimate and heterogeneity. Given the limited change and the number of such cases, all effect sizes were retained, supporting the robustness of the results.

Influence diagnostics results are shown in Figure 3. Removing four effect sizes with an influence of 1 or more yielded g = 0.41 (*z* = 5.53, *p* < 0.01), with a heterogeneity same as the general analysis [Q (52) = 345.81, *p* < 0.01, $I^{2}$ = 85.0, 95%CI (81.1, 88.1), $\tau^{2}$ = 0.24, 95%CI (0.18, 0.49), H = 2.58, 95%CI (2.30, 2.89)]. The prediction interval ranged from −0.58 to 1.39. The removal of influential effect sizes did not significantly alter the results, suggesting robustness. Considering this minimal change and the high number of outliers, this study analyzed all effect sizes.

#### 3.1.3 Subgroup analysis

First, we examined the differences in effect sizes in experimental designs following Harrison and List’s (2004) classification, who considered whether the participants were students or members of the general public as the primary criterion. However, we ignored the participants’ attributes because the purpose was solely to examine the effects of the experimental design. The influence of participants’ attributes has been disentangled and will be analyzed separately at a later stage. Accordingly, we classified the study designs based on the environment: (1) traditional laboratory experiments, (2) framed field experiments (field-related goods, tasks, or information), (3) natural field experiments (similar to 2, but participants were unaware of the experiment), and (4) questionnaire surveys.

Only three effect sizes were classified as framed field experiments, all from the same paper. To avoid overrepresentation by one author, we combined 2 and 3 into “field experiments.”

The difference in effect sizes between subgroups was significant (*p* = 0.02). The effect sizes were the highest in questionnaire surveys (g = 0.51), followed by laboratory experiments (g = 0.43) and field experiments (g = 0.17). The effect diminishes as experimental control decreases, resembling real-world decision-making (Supplementary Table 1). Significant effects were observed only in more controlled environments, such as questionnaire surveys (*p* < 0.01) and laboratory experiments (*p* < 0.01).

More students participated in laboratory experiments, while the general public participated in field experiments; however, they did not fully overlap (Epley et al., 2006; Kim and Tanford, 2021). Subgroup analysis by age group (Supplementary Table 2) showed a moderate effect for students (g = 0.50, *p* < 0.0001) and a low effect for the general population (g = 0.14, *p* < 0.05). The difference in effect sizes was significant (*p* < 0.01), suggesting that the house-money effect was not uniform across age groups.

Regional differences showed no significance (*p* = 0.14); however, the effects were significant in the United States and Asia (*p* < 0.01), with Asia showing a particularly strong effect (g = 0.68, Supplementary Table 3), suggesting that Asians exhibit a stronger house-money effect.

Subgroups based on outcomes (raw expenditure versus other metrics) showed no significant difference in effect sizes (*p* = 0.85), indicating no issues with outcome integration (Supplementary Table 4).

The house-money effect can be divided into two types: regular spending, such as increases in purchasing goods, and risk-taking, such as gambling or investments. Supplementary Table 5 shows the subgroup analysis based on differences in the types of house-money effects being measured. Broadly, cases in which something of equivalent value was always obtained for spending were classified as the former, and others as the latter. Exceptions include the dictator game, classified as the former, as it does not clearly involve risk taking without a reward. The analysis revealed no significant differences in their house-money effect.

Heterogeneity was significant (*p* < 0.01) according to publication year (Supplementary Table 6). The effect sizes decreased over time, with a strong effect before 2009 (g = 0.64) and a weak effect after 2020 (g = 0.18). Earlier studies reported stronger effects, suggesting that robustness may have weakened as the research progressed.

#### 3.1.4 Meta-regression

The meta-regression analysis used variables that showed a certain effect in the subgroup analysis as independent variables: publication year, age-group dummy, research-design dummy, and region dummy (Africa was grouped with Oceania in the Southern Hemisphere). First, a model was created using four variables without considering confounding factors. Additional models assumed confounding factors for the possible variable pairs. A model with confounding factors for all pairs showed $R^{2}$ = 0.64; however, more confounding factors reduced the significance of the permutation test. Therefore, a model without confounding factors was deemed most appropriate, as shown in Table 1. Asian studies showed particularly robust predictors with higher effect sizes.

**Table 1:** Meta-regression results for continuous outcomes.

	Model 1			
	Coeff.	se	*Z*	*p*
Intercept	40.91	20.41	2.00	0.045
Pub year	−0.02	0.01	−1.99	0.047
Age group-general	−0.09	0.20	−0.44	0.6
Method-field	−0.14	0.20	−0.69	0.49
Method-survey	−0.13	0.22	−0.61	0.54
Region-America	0.23	0.26	0.89	0.37
Region-Asia	0.55	0.29	1.91	0.056
Region-Europe	0.11	0.29	0.37	0.71
Obs	57			
$R^{2}$	0.31			
*p*	0.015			
p (permutation)	0.055			

The publication year was the most robust predictor. The model indicates that the effect size decreases by 0.02 with every publication year. This may be related to the time lag bias described by Harrer et al. (2021). Early studies on the house-money effect were groundbreaking and reported notable results. However, subsequent studies scrutinized these findings, potentially mitigating or failing to replicate them. Such critical studies may continue and unpublished evidence potentially denying the house-money effect may persist. To examine these meta-regression findings, we analyzed publication bias.

#### 3.1.5 Publication bias

**Figure 4:**
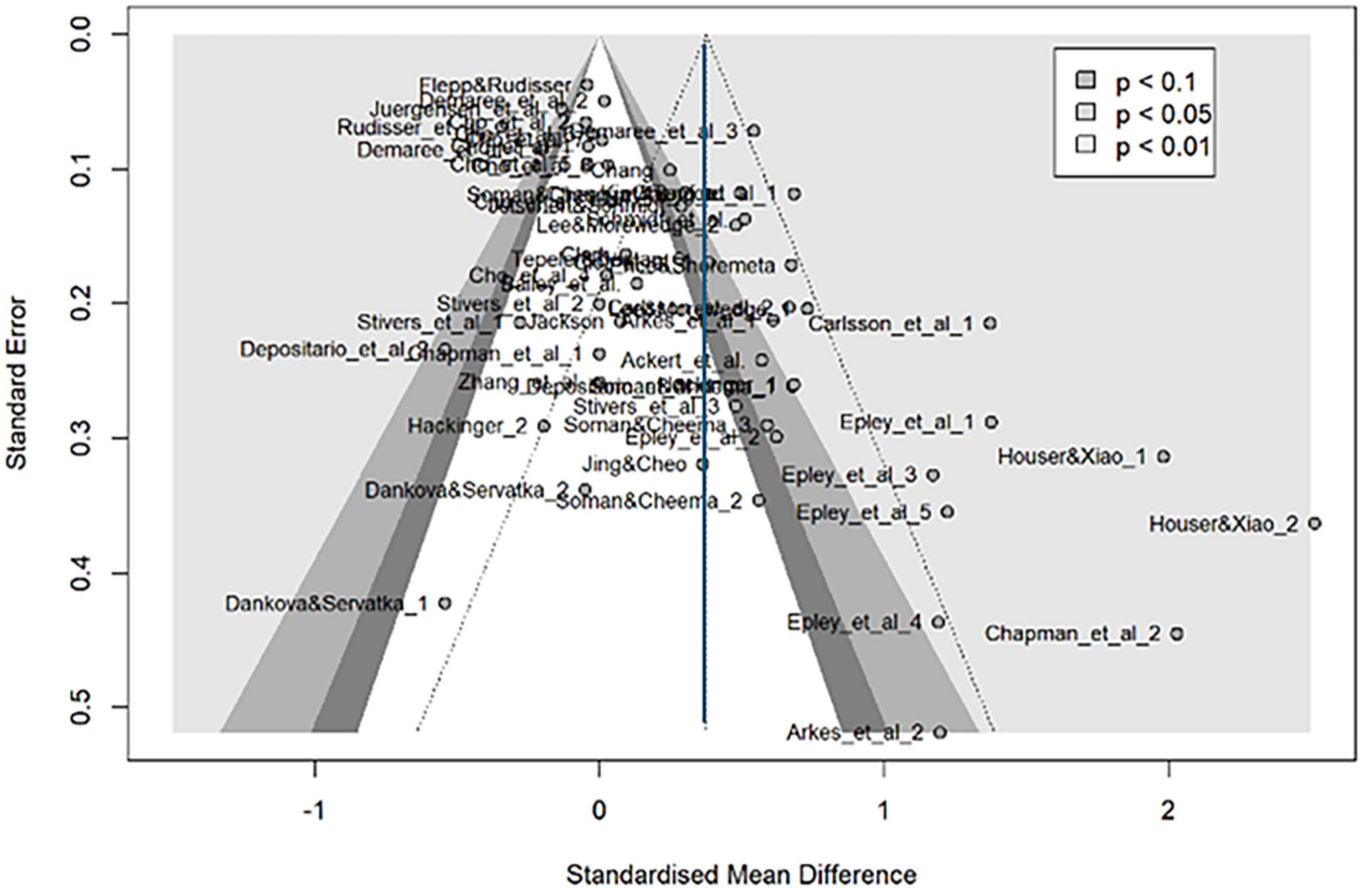
Funnel plot for continuous outcomes. This funnel plot illustrates the relationship between standardized mean differences (x-axis) and standard errors (y-axis) for effect sizes included in the meta-analysis. Each point represents an individual effect size, with its position reflecting its effect size and precision. The shaded areas indicate different levels of statistical significance (*p* < 0.1, *p* < 0.05, and *p* < 0.01). Symmetry in the plot suggests an absence of publication bias, while asymmetry indicates potential bias or heterogeneity. The Trim and Fill method adjusts for missing studies to provide a corrected overall estimate, as reflected in the shaded areas. This funnel plot shows a slight asymmetry, with a tendency for smaller studies to report larger effects. This pattern, supported by Egger’s test, suggests potential publication bias in the study.

**Figure 5:**
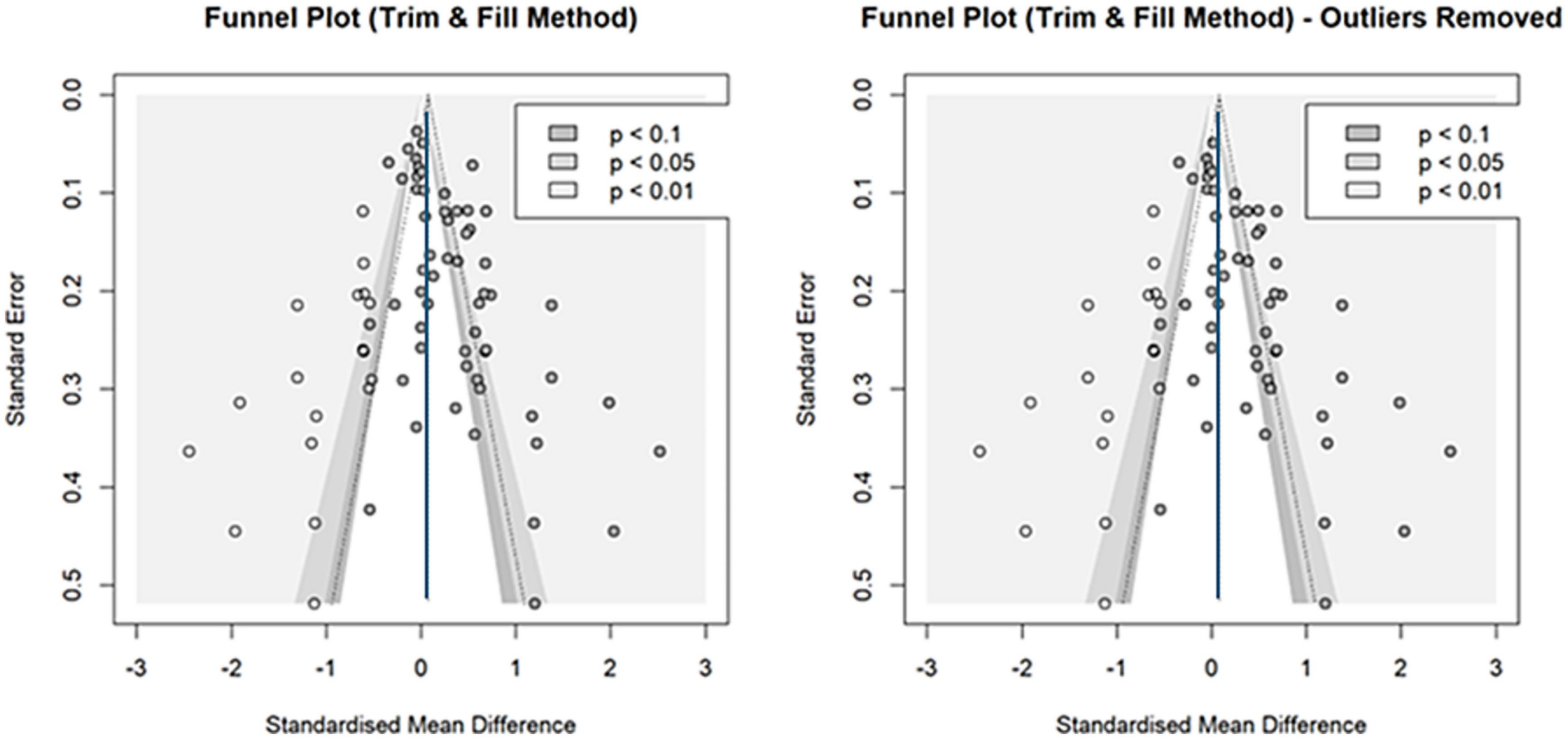
Funnel plots using the Trim and Fill method (continuous outcomes). This figure displays the results of the trim-and-fill analysis. Light-colored dots indicate imputed effect sizes to correct for publication bias. Corrected overall estimates were smaller than the original, suggesting that the house-money effect is likely real but very weak (around g = 0.07).

Figure 4 shows a funnel plot of all the effect sizes, revealing a downward asymmetry to the right, suggesting possible publication bias. Egger test results (Table 2) also indicated asymmetry. To estimate the original effect size, we used the trim-and-fill method to predict the potential studies. The light-colored circles in Figure 5 represent 18 new effect sizes added to the general analysis and 17 effect sizes added to the analysis, excluding the four detected in influence analysis. Both corrected effect sizes were smaller than those in the general analysis [g = 0.07, 95%CI (−0.11, 0.25); g = 0.08, 95%CI (−0.12, 0.27)]. A limit meta-analysis, which predicts effect size from standard error in a regression model (Rücker et al., 2011), calculated an effect size of g = 0.07 [95%CI (−0.13, 0.27)], similar to the above. After correcting for publication bias, the house-money effect was minimal.

**Table 2:** Egger’s test results for continuous outcomes.

	Intercept	95%CI	*t*	*p*
Egger’s test	2.92	1.86–3.98	5.40	< 0.0001

The results of Egger’s test for assessing publication bias in the meta-analysis of continuous outcomes. The intercept value (2.92; 95% CI: 1.86–3.98) indicates significant asymmetry in the funnel plot, suggesting the presence of publication bias.

The *p*-curve analysis (Simonsohn et al., 2014) for p-hacking bias correction (Table 3; Supplementary Figure 4) showed significant right-skewness test results, indicating a positive true effect size and suggesting the existence of an effect. Considering the results of the three publication bias analyses, the general house-money effect is likely not zero but is very weak, around g = 0.07.

**Table 3:** *p*-curve analysis results for continuous outcomes.

	*p* binomial	Full curve		Half curve		Evidential value	
		*z* Full	*p* Full	*z* Half	*p* Half	Present	Absent
Right-Skewness Test	< 0.0001	−12.11	< 0.0001	−10.50	< 0.0001	yes	no
Flatness test	0.99	7.46	1	11.15	1	yes	no

The results of the *p*-curve analysis for continuous outcomes, assessing evidential value and potential publication bias. The “Right-Skewness Test” evaluates whether the *p*-curve is significantly right-skewed, indicating evidential value. The “Flatness Test” examines whether the *p*-curve is flat, suggesting an absence of evidential value. The results show significant right-skewness for both the full and half curves, supporting the presence of evidential value. Flatness tests returned non-significant results, further confirming the robustness of the findings.

### 3.2 Results of binary outcome studies

#### 3.2.1 General meta-analysis

In studies with binary outcomes, risk-seeking and spending behaviors were assessed through forced-choice questions, such as whether to purchase a product or choose a lottery ticket over sure money. This study included 18 effect sizes obtained from the meta-analysis. Risk ratios rather than odds ratios were used for effect sizes, following Harrer et al. (2021). For studies reporting only percentages of house-money-like behavior, raw numbers were estimated from the total sample size and rounded to the nearest integer if needed.

**Figure 6:**
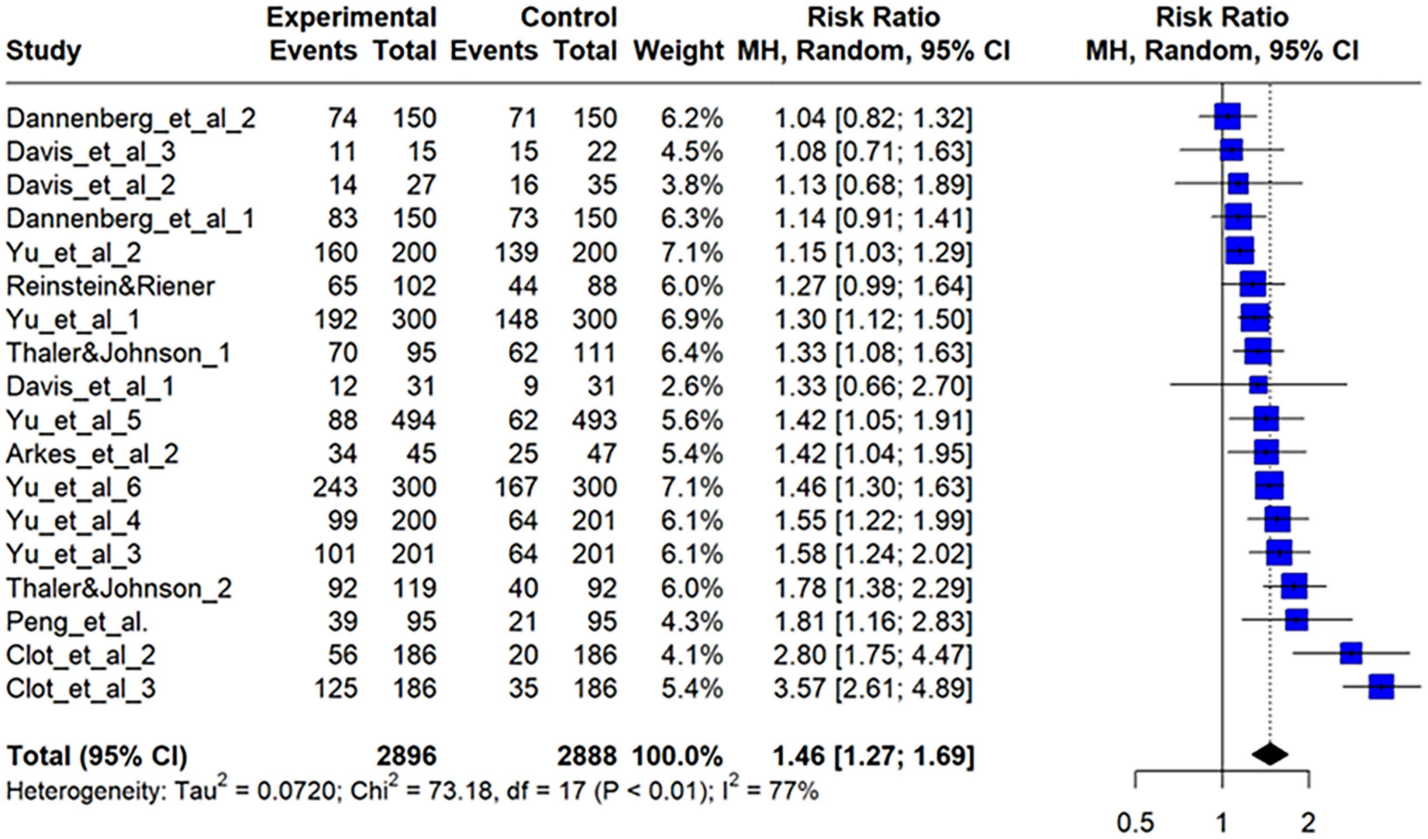
Forest plot for binary outcomes. This forest plot shows the risk ratios (RR) and 95% confidence intervals (CI) for effect sizes included in the meta-analysis. This figure shows the results for 18 effect sizes from studies with binary outcomes, such as risk-seeking behavior in forced-choice decisions. The pooled risk ratio was 1.46, 95% CI [1.27, 1.69], indicating a significant house-money effect. Significant heterogeneity was found (I^2^ = 76.8%), supporting the use of a random-effects model. The prediction interval ranged from 0.81 to 2.63, suggesting both a potential weak reverse house-money effect and a strong effect.

A general analysis pooling all 18 effect sizes yielded a significant rr = 1.46 [95%CI (1.27, 1.69), log rr = 0.38, 95%CI (0.24, 0.52); *z* = 5.19, *p* < 0.01; Figure 6 and Supplementary Figure 5]. Cochran’s Q indicated significant heterogeneity [Q (17) = 73.18, *p* < 0.01], supporting the random-effects model. Other indices also confirmed heterogeneity [ $I^{2}$ = 76.8% > 75, 95%CI (63.6, 85.2%); $\tau^{2}$ = 0.07, 95%CI (0.03, 0.20); H = 2.90 > 1, 95%CI (2.61, 3.21)]. The prediction interval ranged from 0.81 to 2.63 for rr, indicating the possibility of both a slight reverse house-money effect and a strong effect, which exceeded twice the baseline value.

#### 3.2.2 Outliers and influence analysis

Two effect sizes were identified as outliers. Removing them reduced the effect size to rr = 1.33 [95%CI (1.24, 1.44), log rr = 0.29, 95%CI (0.21, 0.36)], although the difference remained statistically significant (*z* = 7.43, *p* < 0.01). This suggests that unexpected income makes people about 1.3 times more likely to choose large expenses and risky options (1.5 times more likely without excluding outliers). Heterogeneity decreased significantly [Q (15) = 26.77, *p* < 0.05, $I^{2}$ = 44.0, 95%CI (0.00, 68.9), $\tau^{2}$ = 0.01, 95%CI (0.00, 0.04), H = 1.34, 95%CI (1.00, 1.79)]. The prediction interval (rr = 1.08–1.65) indicated a reduced likelihood of a reverse house-money effect or a very strong effect.

**Figure 7:**
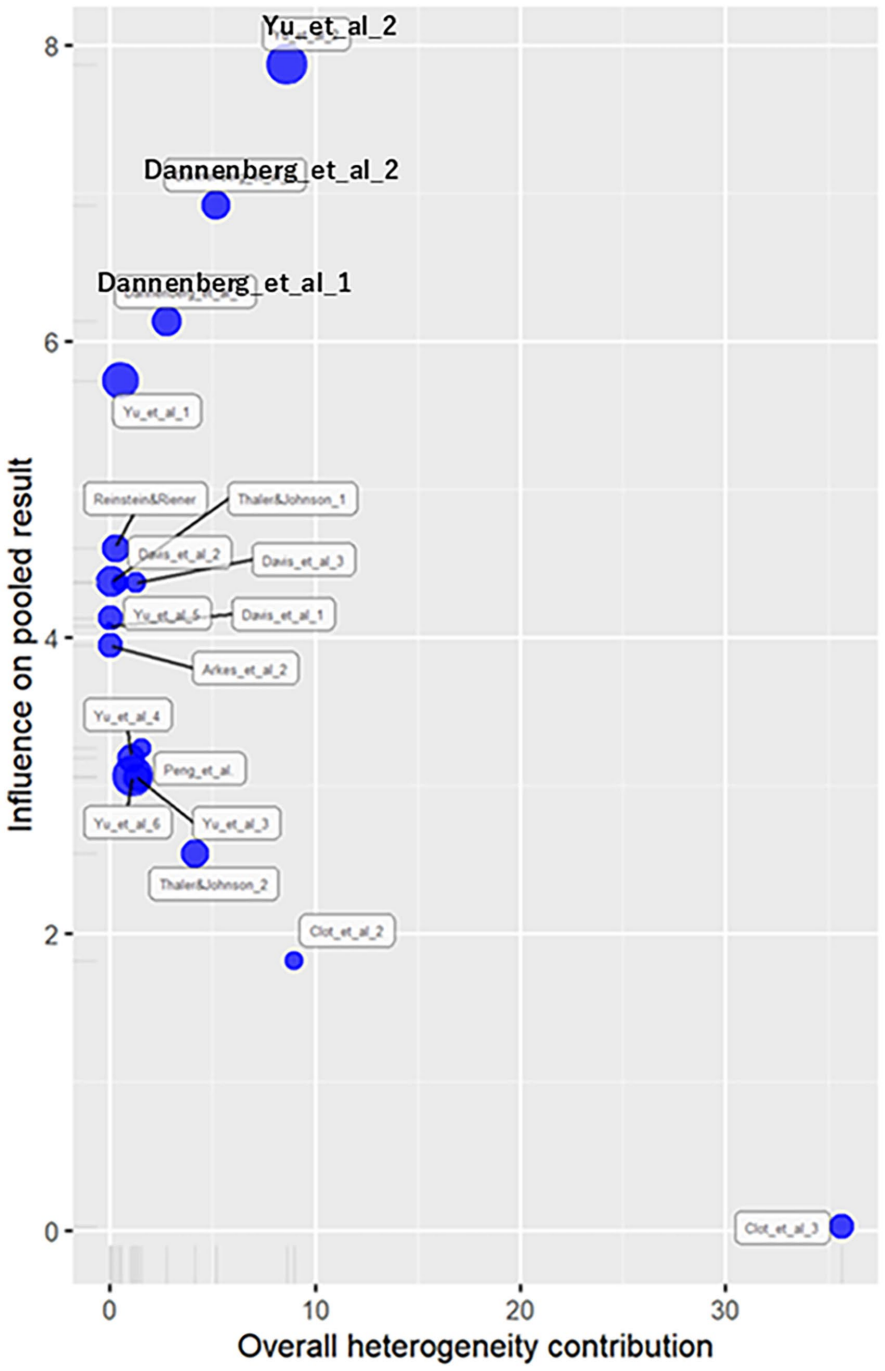
Influence diagnostics plot for binary outcomes. This plot evaluates the influence of individual effect sizes on the pooled meta-analytic result and their contribution to overall heterogeneity. Shows influence values for each binary outcome. After excluding three influential effect sizes, the main result remained robust.

Influence diagnostics (Figure 7) removed three effect sizes with influence ≥ 6, yielding rr = 1.56 (log rr = 0.44), significant at z = 5.57 (*p* < 0.01). Heterogeneity was similar to the general analysis [Q (14) = 49.7, *p* < 0.01, $I^{2}$ = 71.8, 95%CI (52.6, 83.2), $\tau^{2}$ = 0.26, 95%CI (0.15, 0.47), H = 1.88, 95%CI (1.45, 2.44)]. The prediction vginterval of rr ranged from 0.86 to 2.81.

The two outlier effect sizes differed significantly from the others, inflating the pooled effect size, and were excluded from the subsequent analysis. Sensitivity analyses using the maximum-likelihood (rr = 1.33, $\tau^{2}$ = 0.01) and Der Simonian-Laird methods (rr = 1.33, $\tau^{2}$ = 0.01) confirmed consistent results, with $\tau^{2}$ slightly smaller but the same effect size.

After excluding outliers, a three-level meta-analysis was conducted. The results indicated that the two-level random-effects model had better model fit, with lower AIC (−6.48 vs. –8.48) and BIC (−4.48 vs. –7.55) values, suggesting that the simpler two-level structure was more appropriate. The overall effect size estimate was nearly identical between models; in the three-level model, the estimated log risk ratio was 0.29 [95% CI (0.20, 0.37)]. Variance decomposition further showed that 79.2% of the heterogeneity was attributable to between-study variance, indicating that most variation occurred at the study level. Given the robustness of the core results and to avoid unnecessary model complexity, all subsequent analyses were conducted using a standard two-level random-effects model.

#### 3.2.3 Subgroup analysis

The subgroup analysis by research design (Supplementary Table 7) included only laboratory experiments and questionnaire surveys. The difference in heterogeneity was significant (*p* < 0.01) and the effect size was much smaller in the laboratory experiments (rr = 1.14) than in the questionnaire surveys (rr = 1.40).

In the age group analysis, the effect sizes were similar for students (*r* = 1.31, *p* < 0.01) and the general population (rr = 1.35, p < 0.01), with no significant difference in effect size (*p* = 0.68). A consistent house-money effect was observed regardless of age group (Supplementary Table 8).

For regional differences, we grouped regions into America and other countries owing to limited studies to ensure statistical power. No significant heterogeneity was found (*p* = 0.13), and the effect was significant in both groups (Supplementary Table 9).

Combining the effects of increased normal spending or risk poses no issues when considering a single house-money effect (Supplementary Table 10).

Subgroup analysis by publication year (Supplementary Table 11) showed no clear downward trend as observed for continuous outcomes. The heterogeneity was not significant (*p* = 0.10), indicating that the publication year was not a key factor in effect-size variation.

#### 3.2.4 Meta-regression analysis

The subgroup analysis revealed that the research design impacted the effect size. To explore this further, meta-regression analysis was conducted. A model including all four variables, which were also used with continuous outcomes, had adequate explanatory power ( $R^{2}$ = 0.52), but the omnibus test was not significant [QM (4) = 7.91, *p* = 0.09; permutation test: *p* = 0.20]. After testing various models, the best-fit model included only the experimental method dummy variable [ $R^{2}$ = 0.65, QM (1) = 6.37, *p* < 0.01]. Although the full model captured multiple potential moderators, it was not statistically significant, and the inclusion of several interrelated variables raised concerns about multicollinearity, which can obscure the individual contribution of predictors. Moreover, the small sample size in the binary outcome set may have reduced statistical power, increasing the risk of Type II errors. Therefore, the simpler model was preferred for interpretability and parsimony, as it isolated the effect of the most influential variable. The meta-regression results (see Table 4) highlight the strong explanatory power of the experimental method. Unlike the subgroup analysis in continuous outcomes, those with binary outcomes showed little variation between groups, likely because of the overshadowing influence of the research design.

**Table 4:** Meta-regression results for binary outcomes.

	Model 1				Model 2			
	Coeff.	se	*z*	*p*	Coeff.	se	*z*	*p*
Intercept	0.13	0.07	1.93	0.05	0.25	0.12	2.03	0.04
Method-survey	0.19	0.08	2.52	0.01	0.27	0.14	1.84	0.07
Pub year					−0.005	0.01	−0.89	0.37
Age group-general					0.06	0.15	0.41	0.68
Region-America					−0.10	0.14	−0.73	0.47
obs	16				16			
$R^{2}$	0.65				0.52			
*p*	0.01				0.10			
p (permutation)	0.045				0.21			

#### 3.2.5 Publication bias

**Figure 8:**
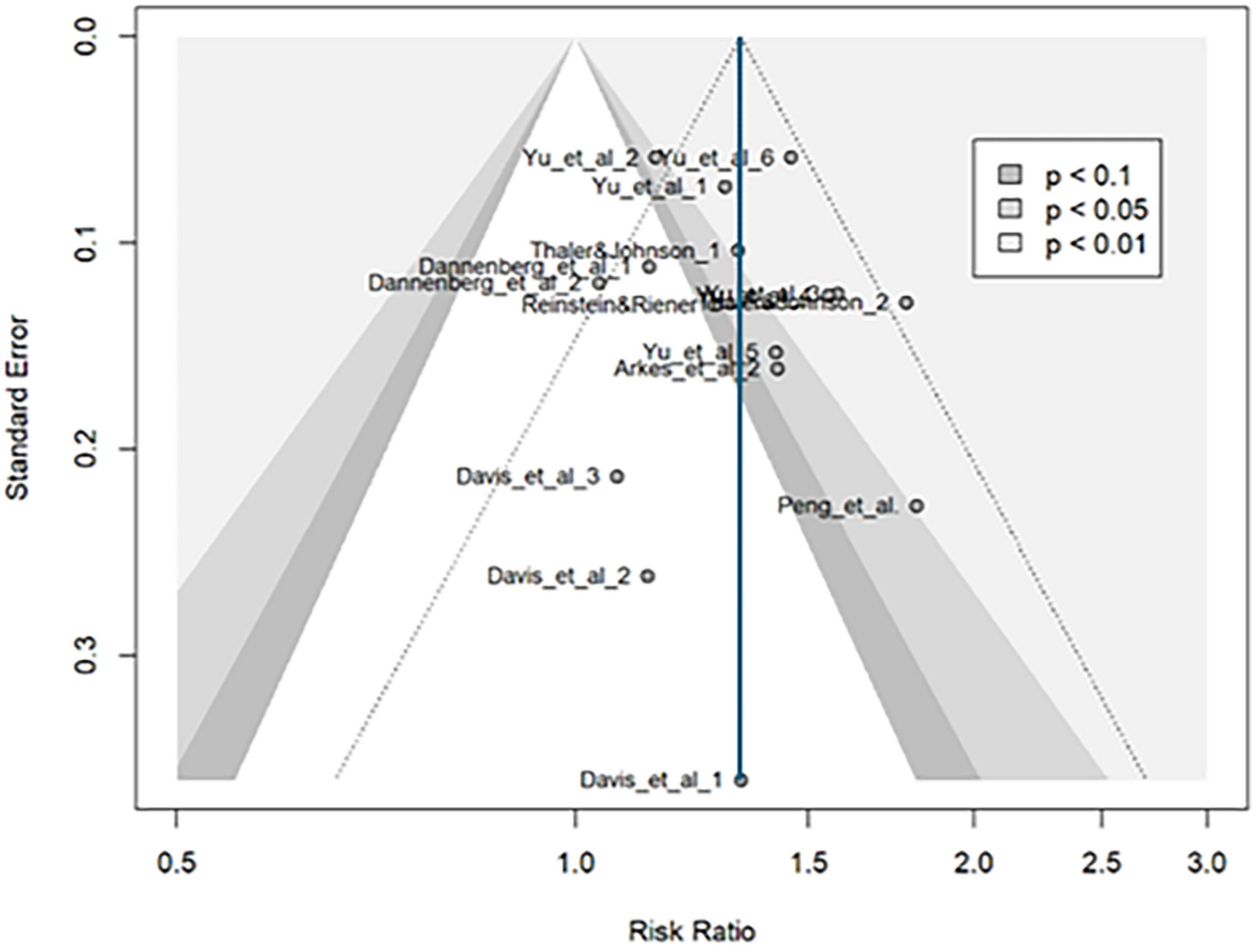
Funnel plot for binary outcomes. The funnel plot was roughly symmetrical. Publication bias analyses (e.g., trim-and-fill) suggested minimal bias, with only slight reductions in effect size.

The funnel plot (Figure 8) was roughly symmetrical, and the Egger test showed no significant publication bias (Table 5). The effect size remains consistent with the general analysis, even after adding one effect size using the trim-and-fill method [rr = 1.32, 95%CI (1.22, 1.42)]. Limit meta-analysis produced a similar effect size [rr = 1.31, 95%CI (1.15, 1.47)]. However, both publication bias corrections slightly reduced the effect size, suggesting a further decrease if more laboratory or field experiments were included.

**Table 5:** Egger’s test results for binary outcomes.

	Intercept	95%CI	*t*	*p*
Egger’s test	0.444	−1.08-1.97	0.57	0.58

The intercept value (0.444; 95% CI: −1.08 to 1.97) indicates no significant asymmetry in the funnel plot, suggesting the absence of publication bias.

The *p*-curve analysis indicated that the true effect size was unlikely to be zero and is positively biased, supporting the existence of a positive effect (Table 6; Supplementary Figure 6). This appears to be a slightly weak to moderate effect, which increases spending and risk-seeking behavior by approximately 1.3 times and being stronger than continuous outcomes.

**Table 6:** *p*-curve analysis results for continuous outcomes.

	*p* binomial	Full curve		Half curve		Evidential value	
		*z* Full	*p* Full	*z* Half	*p* Half	Present	Absent
Right-Skewness Test	0.01	−6.08	< 0.0001	−4.99	< 0.0001	yes	no
Flatness Test	0.97	3.51	1	5.44	1	yes	no

The results show significant right-skewness for both the full and half curves, supporting the presence of evidential value. Flatness tests returned non-significant results, further confirming the robustness of the findings.

## 4 Discussion

Windfall and extra income, such as unearned income, are common in daily life, and the house-money effect has been an accepted theory to explain related behavior. However, the theory’s predictive power remains limited because people’s behaviors are more complex.

The high heterogeneity among studies suggests that the house-money effect varies greatly depending on the context. Separate analyses of continuous and binary outcomes were conducted, including a general meta-analysis, factor investigation, and publication bias analysis. Continuous outcome studies, which were analyzed separately, generally predict increased risk-seeking and spending after windfall income; however, the prediction interval also indicates moderate decreases, suggesting that the theory may not always accurately explain behavior, and could provide incorrect predictions. Subgroup analysis revealed that the house-money effect was more prominent among students and diminished in field experiments. This suggests that the effect may be specific to controlled environments and not be as universal as previously assumed. In natural settings with the general population, this theory lacks explanatory and predictive power.

The year of publication is another key factor in studies on continuous outcomes, with newer studies reporting weaker effects. As the house-money effect faces scrutiny, mitigating factors and limitations have emerged and the reverse house-money effect may gain prominence. Publication bias analysis revealed a significant bias, suggesting that many potential or future studies are skeptical of the effect.

In economics and psychology, the robustness of the effect being studied in laboratory experiments often diminishes in field experiments, a pattern consistent with that of the current study (Gneezy and List, 2006; Benz and Meier, 2008; Falk and Heckman, 2009). These studies also highlighted the partial validity of laboratory experiments. In this study, while the house-money effect decreased significantly when potential effect sizes were considered, it did not reach zero or negative values. Acknowledging the partial validity of laboratory experiments, this study adopted a neutral stance, recognizing the risk of amplifying natural effects. However, this meta-analysis offers new insights into reproducibility and validity issues.

Alternative interpretations are possible beyond the identified issues. As the number of studies increases, people may become more aware of the house-money effect and consciously take rational actions, such as saving windfall income, similar to regular income. Further research is required to confirm this hypothesis.

Despite some negative effect sizes, more than half the effect sizes were positive, confirming the existence of a house-money effect. Although not always significant, this may effectively explain decision-making after windfall income, particularly in Asia and among students, as shown by the subgroup analysis. According to Jagodzinski (2010), life satisfaction increases more with income and living standards in Asia than in Europe. This could explain why the house-money effect is stronger in Asia; people are more satisfied with money and are willing to take risks for further gain and satisfaction.

This, however, raises one question: as participants in lab experiments are more likely to be students, is the lab experiment or students the main explanatory factor? We conclude that both are factors; however, the experimental method plays a larger role. The meta-regression results show that the coefficient of the research design has a greater absolute value, and its influence is overwhelming for binary outcomes as well. Thus, the laboratory setting is more closely related to the strong effect size. However, it is generally believed that people become more risk averse as they age (Gardner and Steinberg, 2005). Therefore, young students can be considered as a risk-seeking group than the general population, clarifying the house-money effect. To further investigate this issue, we tested an interaction model including both study design and participant type. The interaction term was not statistically significant (Coeff. = − 0.26, *p* = 0.15), suggesting that these two factors may contribute independently rather than interactively. Therefore, while student samples and lab settings often co-occur, their effects on the house-money effect appear to be additive rather than synergistic.

In summary, in continuous outcome studies, a mild house-money effect is generally observed; however, it is not strong enough to be universally applicable. Instead, one should carefully consider situations in which the effect does not occur or is even reversed. For binary outcomes, heterogeneity was high, but significantly reduced in the subgroup analysis using the research design. Meta-regression also identified research design as the most important predictor. Binary outcome studies can be categorized into laboratory experiments and questionnaire surveys.

In the general analysis, the effect size was approximately 1.3 times, indicating a weak-to-moderate effect. In laboratory experiments, the effect was weaker by 1.1 times, suggesting a phenomenon unique to controlled environments. If field surveys were conducted, the effect size may diminish further and potentially become negligible. However, unlike the continuous outcome studies, the reverse house-money effect disappeared when outliers were removed in the binary outcome studies, suggesting that it was unlikely to predict opposite behaviors. Instead, unexpected income encourages moderate spending, consumption, and risk-taking. Differences by publication year, age group, and region as well as heterogeneity and publication bias were smaller than those for continuous outcomes. The confidence intervals showed little variation, indicating that the house-money effect was stable and less influenced by context when choosing between two choices.

In summary, when choosing specific options, people behave slightly differently than when spending flexible amounts. Here, the house-money effect provides stable but mild explanatory power. However, the effect size may have been inflated by many questionnaire surveys on binary outcomes. Adding field-like experiments could have revealed patterns similar to those of the continuous outcomes.

The house-money effect was observed in both cases, continuous and binary outcome studies, but remained very mild in certain analyses such as publication bias and so on. Though individuals such as Asians, students, and participants in controlled environments tend to be more sensitive to the psychological categorization of money based on its source, mental accounting may also have its limitations.

Though the tendency of windfall income to reduce spending and increase risk aversion was termed the “reverse house-money effect” for convenience, future research should integrate this phenomenon into new theories within behavioral economics and decision-making. As a possible explanation for the reversed or modest house money effect, loss aversion may play a key role and should be further investigated in future research.

As an example of the application of this study’s findings, the tendency of students to be easily influenced can inform systems such as tuition exemptions and scholarships. Emphasizing a “regular tuition refund” rather than a “bonus” may reduce impulsive spending. However, for the general population, the possibility of a reverse house-money effect becomes more likely. Thus, counterintuitively, labeling part of a salary as a “bonus” may effectively promote everyday savings, while calling it “regular salary” could encourage consumption. Offering discounts or “point refunds” as windfall income can also be effective. A stable effect may be achieved by using online ordering methods that resemble questionnaire surveys or by providing specific product options rather than traditional in-store purchases. However, because the house-money effect is not robust or strong, it should be combined with other measures to maximize its impact.

### 4.1 Limitations

This study also has some limitations. First, the lack of individual risk of bias assessments is a limitation of the current research process. However, although a formal risk of bias tool was not applied, this meta-analysis follows a strictly quantitative framework, inherently excluding studies that lack the numerical clarity necessary for reliable estimation of effect sizes. As a result, studies that are arbitrary or methodologically opaque are unlikely to be included, and nearly all included studies were published in high-quality peer-reviewed journals ranked Q2 or higher. Additionally, as described in the Methods section, only between-subjects experiments with appropriate control groups were included, following the PICO framework. A screening of the full texts confirmed that most studies clearly reported random assignment and hypothesis-driven designs. Taken together, although the absence of formal scoring remains a limitation, the included studies are likely to possess high internal validity.

Furthermore, this study has limitations owing to the heterogeneity of the research groups, which combine potentially distinct phenomena. Social science experiments often show large variations owing to subtle differences (Tversky and Kahneman, 1974). While this study focused on general trends at the expense of subtle factors, it revealed limitations in the widely accepted explanations. Future research on the limitations of the house-money effect should focus on the following key areas. First, boundary conditions should be identified, as it is crucial to understanding where the effect occurs, such as cultural or contextual factors, incentive size, and experimental settings. Second, if the house-money effect is not observed, alternative mechanisms like risk aversion, mental accounting, or interactions with other biases must be examined to explain decision-making in its absence. Third, individual differences, such as demographic factors, psychological traits, and group-specific behaviors (e.g., consumers vs. investors), should be analyzed. Regarding long-term impacts, this study’s findings suggest that the house-money effect is weak; this implies that even in cases where the effect is initially strong, it may fade over time. This highlights the need for temporal studies to investigate how the effect evolves or disappears with time. Furthermore, cross-cultural and economic system comparisons could clarify how the effect varies globally. Finally, innovative methods like neuroeconomics, big data, and computational modeling can deepen insights into mechanisms, operating when the effect is absent or limited. These directions are vital for advancing our understanding of financial decision-making.

### 4.2 Conclusion

This study reveals that the widely accepted house-money effect is, in fact, a theory with significant limitations. These findings offer insights into effective policy design and consumer behavior. In addition, this study highlights under-researched areas and suggests future directions to advance this field. Moreover, this study suggests the potential limitations of mental accounting as an explanatory framework. Future research should explore this issue more extensively across a wider range of contexts. Beyond offering a summary of existing findings, this meta-analysis provides a structured and theory-informed synthesis of previously fragmented results. By systematically organizing decades of heterogeneous evidence and applying a multi-layered analytical strategy—including outlier analysis, three-level modeling, and publication bias correction—it contributes a coherent perspective to a field that has lacked integrative clarity.

## Data Availability

The original contributions presented in the study are included in the article/Supplementary material, further inquiries can be directed to the corresponding author.
